# The effect of the waiting room’s environment on level of anxiety experienced by children prior to dental treatment: a case control study

**DOI:** 10.1186/s12903-019-0995-y

**Published:** 2019-12-30

**Authors:** Avia Fux-Noy, Maayan Zohar, Karin Herzog, Aviv Shmueli, Elinor Halperson, Moti Moskovitz, Diana Ram

**Affiliations:** Department of Pediatric Dentistry, Hadassah School of Dental Medicine, P.O.Box 12272, 9112102 Jerusalem, Israel

**Keywords:** Waiting room, Dental anxiety, Multisensory environment

## Abstract

**Background:**

In addition to visit purpose, one of the environmental factors that can cause anxiety prior to dental treatment includes the waiting room experience, specifically the amount of time spent awaiting treatment and the waiting room environment. The purpose of this study was to compare the effect of the waiting room’s environment on the level of anxiety experienced by children in multisensory and traditional waiting rooms.

**Methods:**

Case control study. Test group waited for treatment in a multisensory waiting room, which consisted of a lighting column that children could touch and climb; as well as, rhythmic music played on loudspeakers. Control group waited for treatment in a traditional waiting room. Study participants were asked to answer the “Venham Picture Test”, a dental anxiety scale, while in the waiting room prior to entering the treatment room. Chi-squared, Fisher’s Exact tests, and linear regression were utilized. A *p-*value less than 0.05 was considered statistically significant.

**Results:**

No significant difference in dental anxiety scores was found between the test and control groups according to waiting room type (*p* > .05). Dental anxiety was significantly higher in patients who had longer waiting time prior to treatment (*p* = 0.019). In addition, dental anxiety was significantly associated with visit purpose (*p* < .001): children waiting for dental examination or those scheduled for dental treatment with conscious sedation were less anxious than children waiting for emergency treatment.

**Conclusions:**

A sensory adapted waiting room environment may be less important in reducing children’s anxiety prior to dental treatment. Children’s dental anxiety can be reduced by preventing emergency treatments, scheduling routine dental visits and decreasing waiting time.

**Trial registration:**

TRN NCT03197129, date of registration June 20, 2017.

## Background

It has been estimated that about 11% of children and adolescents suffer from dental anxiety [1]. The level of the subject’s dental anxiety is affected by environmental factors and personality traits [2]. One of the environmental factors that can cause anxiety prior to dental treatment includes the waiting room experience [3], specifically the amount of time spent awaiting treatment and the waiting room environment [4]. Existing literature addressing the waiting room environment and atmosphere evaluated different methods to reduce anxiety in the waiting room, such as exposure to positive images of dentistry [5, 6], aromatherapy [7, 8], and music [9–11].

The Snoezelen environment, which consists of a multisensory adapted environment coupled with client-centered therapy, provides a soothing atmosphere for patients with cognitive impairment [12]. The sensory adapted dental environment, developed based on the Snoezelen environment, has been found to significantly reduce dental anxiety and maladaptive behaviors, while facilitating a calming effect in the dental clinic among children during routine dental prophylactic cleaning [13–15]. While this technique has been studied in the operating room during operative treatment, the impact of waiting in a multisensory adapted environment on anxiety prior to dental treatment has not been evaluated. Hence, the aim of this study was to compare the effect of multisensory and traditional waiting room environments on the level of dental anxiety experienced by children prior to dental treatment.

## Methods

### Study population

Participants were recruited from the pediatric dental clinic at Hadassah Ein Kerem. Inclusion criteria: males and females between 3 and 10 years of age, who were waiting for a dental examination or dental treatment. Exclusion criteria: patients who have developmental disorders and were mentally or cognitively unable to understand or answer the Venham Picture Test (VPT) [16] that assesses anxiety before treatment; as well as, patients accepted immediately on arrival for their dental visit without spending time in the waiting room. Written informed consent was obtained from all parents/guardians of the participants included in the study.

### Study design

This is a case control study. The test group was waiting for dental treatment in the multisensory waiting room: a small room (2.5mx2.5 m) located inside the pediatric dental clinic, with 6 seats. The multisensory environment consisted of a lighting column that children could touch and climb on. Auditory stimuli included rhythmic music, played on loudspeakers. The control group waited for dental treatment in the traditional waiting room, located in the dental school lobby outside the pediatric dental clinic. This waiting room consisted of ten seats in one row facing the reception desk, and was air-conditioned, well-lit, without posters or paintings on the walls, and no reading material. Since the test group’s waiting room was closer to the operating rooms, it is possible that noise from the operating rooms was heard in the test group’s waiting room. However, music was played in the test waiting room, which may have concealed these sounds. Participants were randomly assigned to the test or control group by a toss of a coin.

Participants were asked to answer the VPT, a dental anxiety scale, while waiting in the waiting room just before entering the treatment room. VPT is widely used and easily administered. In this test, children were presented with eight cards, with two figures on each card: one anxious figure and one non-anxious figure. The main investigator, wearing a white dental uniform, asked the children to choose the figure from each pair that described how they felt at that particular time. All cards were shown in their numbered order. If an anxious figure was chosen, a score of one was recorded. If a non-anxious figure was chosen, a score of zero was recorded. A measure of anxiety was obtained by totaling the number of times the child picked the figure depicting the anxious state (minimum score 0; maximum score 8).

Additional data including gender, age, waiting time, purpose of visit, parent/guardian accompanying child, child’s dental history and experience was collected from the parents by the main investigator.

### Sample size and power calculation

A pilot study was conducted with 40 subjects per study group. Based on this study, a power analysis was completed to determine an appropriate sample size. To achieve a significance level at the 95th percentile confidence level and a power of 80%, with a 0.5 estimated effect size, the sample size was calculated to be 51 subjects in each group.

### Ethical considerations

Study protocol was approved by the Institutional Human Subjects Ethics Committee of Hadassah Medical Organization IRB, Jerusalem, Israel. All procedures performed were in accordance with the ethical standards of the institutional and national research committee. The study protocol was also enrolled, and the full trial protocol can be accessed at clinicaltrials.gov (NCT03197129). Detailed information in simple non-technical language was provided in advance and parents/guardians of all patients included in the study were requested to sign an informed consent. No compensation was provided for participating patients.

### Statistical analysis

Data analysis was performed using statistical software (Stata version 12.1, StataCorp). Descriptive statistics were tabulated for the demographic and clinical characteristics. Chi-squared and Fisher’s Exact tests were utilized to test the association between the categorical variables and waiting room type. A T test with unequal variance was used to test the association between VPT score and waiting room type. We estimated unadjusted and adjusted slopes from linear regression to examine the association between VPT score and waiting time; as well as, waiting room type. For this analysis, a *p-*value less than 0.05 was considered statistically significant.

## Results

One hundred twenty-two children were recruited for this study. Nine dropped out since they did not answer the VPT (due to shyness or unwillingness). One hundred and thirteen children participated in the study: 61 males (54%) and 52 females (46%). Age range was 3–10 years, with an average age of 5.7 years (SD = 2). The test group comprised of 56 children and the control group comprised of 57 children. Demographic and clinical characteristics of the groups are presented in Table 1. There was a significant difference between the test and control group in regards to visit purpose (Fisher’s Exact, *p* < .001): more children in the test group waited for dental treatment with oral conscious sedation, while more children in the control group waited for a dental examination or emergency treatment. There was no significant difference in gender, age, waiting time, dental history, dental experience or patient’s escort between the two groups (Table 1).

**Table 1:** Demographic and clinical characteristics by waiting room type

		Total (*N* = 113)		Test Group Multisensory Waiting Room (*N* = 56)		Control Group Conventional Waiting Room (*N* = 57)		Test statistic, *P*-value
		Freq.\ Mean	SD\%	Freq.\ Mean	SD\%	Freq.\ Mean	SD\%	
Gender	Male	61	54%	33	59%	28	49%	Χ^2^(1) = .798, *p* > .05
	Female	52	46%	23	41%	29	51%	
Age (years)		5.68	1.98	5.63	1.87	5.73	2.1	t(95) = 0.28, *p* > .05
Waiting length (minutes)		20.51	22.11	22.43	26.5	18.63	16.75	t(95) = −.909, *p* > .05
Visit purpose	Examination	21	19%	0	0%	21	37%	Fisher’s Exact, *p* < .001
	Emergency	13	11%	1	2%	12	21%	
	Treatment- no sedation	4	3.5%	2	4%	2	3.5%	
	Treatment with preoperative sedation- midazolam	32	28%	23	41%	9	16%	
	Treatment with preoperative sedation- atarax	36	32%	25	45%	11	19%	
	Treatment with sedation- N_2_O	4	3.5%	2	4%	2	3.5%	
	Treatment with preoperative sedation- valium	3	3%	3	5%	0	0%	
Dental history	None	4	4%	1	2%	3	5%	Fisher’s Exact, *p* > .05
	Examination only	26	23%	12	21%	14	25%	
	Treatment	83	73%	43	77%	40	70%	
Dental experience	Positive	60	53%	34	61%	26	46%	Fisher’s Exact, *p* > .05
	Negative	49	43%	21	37%	28	49%	
	NA	4	4%	1	2%	3	5%	
Escort	Mother	58	51%	31	55%	27	47%	χ^2^(1) = 1.293, *p* > .05
	Father	39	35%	19	34%	20	35%	
	Both	16	14%	6	11%	10	18%	

There was no significant difference in mean VPT scores between the two groups with regards to room type (t (95) = 0.668, *p* > .05) (Table 2). In order to overcome the difference in visit purpose, further analysis evaluating the subset of patients only treated with conscious sedation found no significant difference in mean VPT scores between groups (t (53) = .346, *p* > .05) (Table 2). There was a significant association between VPT scores and visit purpose (F (3,109) = 5.03, *p* < .001). Mean VPT scores and visit purpose are presented in Table 3. Those who presented for an emergency/pain visit had significantly higher VPT score (*P* < 0.001). The unadjusted linear regression analysis found a significant association between VPT score and waiting time, with a longer waiting time associated with higher VPT score (*p* = 0.019). This association was still significant even after adjusting by room type, age and gender (*p* = 0.013). There was no association between VPT score and type of waiting room (Table 4). Mean VPT scores and waiting time are presented in Table 5.

**Table 2:** VPT scores according to waiting room type

VPT score	Groups			
	Test *N* = 56	Control *N* = 57	Test- sedation only *N* = 53	Control- sedation only *N* = 22
	Freq (%)	Freq (%)	Freq (%)	Freq (%)
0	26 (46.43%)	22 (38.60%)	24 (45.28%)	11 (50%)
1	6 (10.71%)	8 (14.04%)	6 (11.32%)	3 (13.64%)
2	11 (19.64%)	9 (15.79%)	11 (20.75%)	3 (13.64%)
3	4 (7.14%)	3 (5.26%)	4 (7.55%)	0
4	0	7 (12.28%)	0	0
5	4 (7.14%)	2 (3.51%)	4 (7.55%)	2 (9.09%)
6	1 (1.79%)	3 (5.26%)	1 (1.89%)	0
7	2 (3.57%)	1 (1.75%)	1 (1.89%)	1 (4.55%)
8	2 (3.57%)	2 (3.51%)	2 (3.77%)	2 (9.09%)
Mean (SD)	1.71 (2.27)	2 (2.28)	1.68 (2.19)	1.91 (2.78)
T-Test	t(95) = .668, *p* > .05		t(53) = .346, *p* > .05

**Table 3:** Mean VPT scores and visit purpose

Visit purpose	Mean VPT (SD)		
	Total (*N* = 113)	Test Group (*N* = 56)	Control Group (*N* = 57)
Examination	1.14 (1.56)	NA	1.14 (1.56)
Emergency	3.92 (1.66)	7	3.67 (1.44)
Treatment- no sedation	1 (2)	0	2 (2.83)
Treatment with preoperative sedation- midazolam	1.94 (2.66)	1.61 (2.15)	2.78 (3.70)
Treatment with preoperative sedation- atarax	1.78 (2.23)	1.96 (2.35)	1.35 (1.96)
Treatment with preoperative sedation- valium	1 (1.73)	1 (1.73)	NA
Treatment with inhaled sedation- N_2_O	0.5 (1)	0	1 (1.41)
Overall mean (SD)		1.71 (2.27)	2 (2.28)
T-Test		t(95) = .668, *p* > .05

**Table 4:** Associations between covariates and VPT scores using unadjusted and adjusted linear regression

		Unadjusted		Adjusted	
		Slope	*P*-value	Slope	*P*-value
Waiting room type	Conventional	reference	NA	reference	NA
	Multisensory	−0.29	0.505	−0.46	0.272
Age		−0.16	0.133	−0.17	0.099
Waiting time (minutes)		0.23	0.019	0.24	0.013
Gender	Male	reference	NA	reference	NA
	Female	−0.59	0.167	− 0.68	0.108

**Table 5:** Mean VPT scores and waiting time

Waiting time (minutes)	Mean VPT (SD)		
	Total (*N* = 113)	Test Group (*N* = 56)	Control Group (*N* = 57)
< 10	1.92 (2.51)	1.8 (2.29)	2.14 (2.93)
10–19	1.03 (1.36)	0.67 (1.07)	1.23 (1.48)
20–29	1.86 (2.73)	3 (3.61)	1 (2)
30–39	3 (1.77)	1.5 (2.12)	3.5 (1.52)
40–49	2.5 (2.17)	0.5 (0.71)	3.5 (1.91)
50–59	1.5 (2.38)	0	2 (2.65)
> 60	2.93 (2.95)	2.73 (2.80)	3.67 (4.04)
Overall mean (SD)		1.71 (2.27)	2 (2.28)
T-Test		t(95) = .668, *p* > .05	

## Discussion

Nonverbal communication is a behavior management technique used by pediatric dentists [17, 18]. A child-friendly atmosphere in the clinic is one aspect of this technique. Jayakaran et al. [19] evaluated the pediatric dental operatory environment and found that cartoon-painted walls, toys, and a scented environment reduced anxiety in children. In this current study we focused on the pediatric dental waiting room environment. We found no significant difference in the anxiety of children waiting for dental treatment in a multisensory waiting room or conventional waiting room.

Coffey and Di Giusto [4] also found no difference between anxiety scores of patients in two different dental hospitals with different waiting room environments. In the Coffey and Di Giusto study, the test environment aimed to increase patient ease and relaxation by including comfortable padded seats, reading material for patients, large windows overlooking a garden, and piped music played at low volume. However, this study was conducted on an adult population, and excluded patients requiring a tooth extraction or those who had other stress-inducing problems. Our study, on the other hand, included participants regardless of their visit purpose.

In regards to the pediatric population, it has been suggested that waiting rooms designed for children can reduce their anxiety. Studies have found that positive dental images reduce anxiety compared with neutral images [5, 6]. In addition, viewing positive images of dentistry and dentists was correlated with short-term reductions in anticipatory anxiety in children [5, 6]. Panda et al. [20] evaluated 212 children between 6 and 11 years of age, and found that majority of children preferred music and the ability to play in a waiting room. They also preferred natural light and walls with pictures. Children favored gazing at an aquarium or watching television; as well as, sitting on beanbags and chairs. They were fond of plants and oral hygiene posters [15].

In our study, anxiety was found to be correlated with visit purpose. Children waiting for dental examination or those scheduled for dental treatment with conscious sedation were less anxious than children waiting for emergency treatment. Since groups differed by visit purpose it is difficult to conclude about the effect of waiting environment according to visit purpose. It can be expected that children waiting for treatment after receiving a dose of premedication will be less anxious prior treatment as a result of the medication. Children presenting for dental check-up should be less anxious than those who are aware of the need for dental treatment due to pain or another emergency. Peretz and Kharouba [21] reported high dental anxiety among patients who expected operative procedures. Soares et al. [22] also found differences in dental anxiety in children associated with the visit purpose when analyzing three treatment types: preventive care, endodontic treatment, and dental extraction. Coffey and Di Giusto [4] reported that anxiety levels were higher in adult patients who presented for the first time than in those who came for a subsequent visit [4]. These findings emphasize the need for establishing a dental home, having periodic check-ups, and scheduling routine dental visits; thus, it is proposed that preventing the need for emergency dental treatment in an unfamiliar environment can reduce children’s dental anxiety.

This study found that longer waiting time was associated with higher anxiety. This finding corroborates previously published literature showing that anxiety levels are significantly higher in dental patients who waited for longer periods of time [4, 23]. Dentists must make the effort to shorten the waiting time as possible. In contrast to other studies [24, 25], the current study did not find any relation between age or gender and dental anxiety, perhaps because it tested anxiety while waiting for treatment and not dental anxiety in general.

This study was limited by the low overall anxiety in the study group, based on the low VPT scores. This limitation may have contributed to the non-significant findings in anxiety scores between patients treated in different waiting room environments. It is possible that conducting the study in a population with higher levels of anxiety would have different results. Another limitation was the difference in visit purpose between groups that may have affected the results and the ability to compare between them. In addition, due to the nature of the intervention; two different waiting areas with different designs, the participants and the examiner could not be blinded to the intervention. Although there was a potential for bias, it did not influence the results of this study as we found no significant difference in the anxiety of children.

In view of the limitations of the current study, future research should include a more heterogenous sample of anxiety levels and more homogeneity in visit purpose, such as comparing patients who present for similar types of dental procedures.

## Conclusions

A sensory adapted waiting room environment may be less important in reducing children’s anxiety prior to dental treatment. There was no difference according to waiting room type; however visit purpose and waiting time had a significant effect on dental anxiety scores. Children’s dental anxiety can be reduced by preventing emergency treatments, scheduling routine dental visits and decreasing waiting time.

## Data Availability

Raw data is available in supplemental material.

## Abbreviations

VPT: Venham Picture Test
